# The Association Between Microalbuminuria and QTc Prolongation in Patients With Type 2 Diabetes Mellitus: A Single-Centre Study From South India

**DOI:** 10.7759/cureus.35646

**Published:** 2023-03-01

**Authors:** Solomon K Dominic, Renoy A Henry, Niveditha Kartha, Gopalakrishna Pillai

**Affiliations:** 1 Internal Medicine, Amrita Institute of Medical Sciences and Research Center, Kochi, IND; 2 Biostatistics, Amrita Institute of Medical Sciences and Research Center, Kochi, IND

**Keywords:** south india, diabetic nephropathy, microalbuminuria, prolonged qtc interval, type 2 diabetes mellitus

## Abstract

Background: One of the most significant complications of type 2 diabetes mellitus (T2DM) is diabetic nephropathy, the leading cause of end-stage renal disease. Another important clinical marker in patients with type 2 diabetes is QTc interval prolongation. We aimed to study the association between QTc interval prolongation and microalbuminuria in patients with T2DM.

Objective: The primary objective of this study was to examine the association between QTc interval prolongation and microalbuminuria in patients with T2DM. The secondary objective was to correlate the prolongation of the QTc interval with the duration of T2DM.

Materials and methods: This study was conducted as a single-centre, prospective, observational study in a tertiary-care centre in South India, Amrita Institute of Medical Sciences and Research Center. The study was conducted over two years, between April 2020 and April 2022. Patients aged more than 18 with T2DM with and without microalbuminuria were recruited into the study and control groups, and various parameters, including QTC intervals, were recorded.

Results: A total of 120 patients were enrolled in the study, with 60 patients with microalbuminuria forming the study group and 60 patients without microalbuminuria forming the control group. There was a statistically significant association between microalbuminuria with a prolonged QTc interval, hypertension, a longer duration of T2DM, higher haemoglobin AIc (HbA1c) levels, and higher serum creatinine values.

## Introduction

Type 2 diabetes (T2DM) is a chronic metabolic disorder characterised by hyperglycaemia resulting from insulin resistance and insufficient insulin secretion. It is a major public health problem affecting an estimated 463 million people worldwide, with prevalence projected to rise to 578 million by 2030 [1]. India has one of the highest diabetes burdens in the world, with an estimated 77 million adults living with diabetes in 2019 [2].

One of the most significant complications of T2DM is diabetic nephropathy, the leading cause of end-stage renal disease (ESRD) worldwide. Diabetic nephropathy is associated with increased morbidity and mortality and poses a significant economic burden to the healthcare system [3]. In India, the prevalence of diabetic nephropathy in patients with T2DM ranges from 20% to 27%, with rates increasing with increasing duration of diabetes [4,5]. Early detection and treatment of diabetic nephropathy are critical to prevent or delay the onset of ESRD and reduce the overall burden of the disease.

Another important clinical marker in patients with type 2 diabetes is QTc interval prolongation. QTc prolongation is influenced by several factors, including medication, electrolyte imbalance, and genetic factors. The prevalence of QTc prolongation in patients with type 2 diabetes was reported to be as high as 44%, with higher haemoglobin AIc (HbA1c) levels and microalbuminuria being found to be independent risk factors for QTc prolongation [6,7]. Regular electrocardiogram (ECG) screening for QTc prolongation in patients with type 2 diabetes can help identify individuals with an increased risk of cardiovascular events and guide appropriate intervention.

As T2DM is a major public health problem in India, with a high incidence of diabetic nephropathy and reported QTc interval prolongation in South India, early detection and treatment of these complications is essential to prevent or slow down disease progression and reduce the burden of disease on patients and healthcare systems. We aimed to study the association between QTc interval prolongation and microalbuminuria in patients with T2DM from a tertiary-care centre in South India.

## Materials and methods

This study was conducted as a single-centre, prospective, observational study in a tertiary care centre in South India, Amrita Institute of Medical Sciences and Research Center. The study was conducted over two years between April 2020 and April 2022. The primary objective was to examine the association between QTc interval prolongation and microalbuminuria in patients with T2DM. The secondary objective was to correlate the prolongation of the QTc interval with the duration of T2DM. Ethical committee clearance was received from the institutional review board (ECASM-AIMS-2022-250).

Sample size calculation

Based on the prolonged-QTc-interval in diabetics with normoalbuminuria and patients with microalbuminuria, an odds ratio of 4.3, as observed in a study by Mittal et al. [8], with 90% power and 95% confidence, the minimum sample size was calculated to be 47 in each group, with a total of 94 patients. A total of 120 patients were enrolled in the study.

Inclusion and exclusion criteria

Patients were included in the study if they were more than 18 years of age and had T2DM. They were excluded if they were previously diagnosed with ischemic or structural heart disease, cardiomyopathy, heart block, or heart failure, had any arrhythmias, were on drugs that cause QTc prolongation or type 1 diabetics, had underlying thyroid disorders, had an infection at the time of QTc measurement, had a chronic kidney disease with an alternate pathology, or had dyselectrolytaemia.

All patients underwent a history and physical examination and biochemistry tests for renal function, serum HbA1C, and urine albumin.

Definitions

T2DM was defined as ‘FBS ≥ 126 mg/dl or random blood sugar (RBS) ≥ 200 mg/dl or HbA1c ≥ 6.5 % or an oral glucose tolerance testing showing a two-hour plasma glucose value ≥ 200 mg/dl’ [8].

Diabetic nephropathy is a progressive kidney disease that is a complication of diabetes. It is characterised by the presence of albumin in the urine (albuminuria) and decreased kidney function. Diabetic nephropathy was defined as ‘albuminuria greater than 30 mg per 24 h or a urinary albumin-to-creatinine ratio (ACR) greater than or equal to 30 mg/g or as decreased estimated glomerular filtration rate (eGFR) defined as eGFR <60 mL/min/1.73 m2 for ≥3 months’ [9]. Microalbuminuria was defined as a urinary albumin excretion rate (UAER) of 30 to 300 mg/g [10].

QTc interval prolongation is a measure of the duration of ventricular repolarisation on the ECG. This was defined as ‘a QTc interval greater than 440 ms in males or 460 ms in females’ [11].

Statistical analysis

Statistical analysis was done using IBM SPSS Version 20 (IBM Corp. Released 2020. IBM SPSS Statistics for Windows, Version 27.0. Armonk, NY: IBM Corp). Categorical variables were expressed as percentages and frequencies. Continuous variables were expressed as mean and standard deviation or as a median. For categorical variables, a chi-square test or Fischer’s exact test was used. For continuous variables, unpaired T-tests were used for normally distributed data, while a Mann-Whitney U test was used for non-normally distributed data. If the p-value was less than 0.05, it was considered significant.

## Results

The total number of patients who were enrolled in the study was 120. There were 60 (50%) patients with microalbuminuria (study group) and 60 (50%) patients without microalbuminuria (control group).

The mean age in the study was 58 ± 11.1. The mean age in the study group was 59.9±12.2, while the mean age in the control group was 56.2±9.6. There was no statistically significant association identified between age and microalbuminuria (p=0.065). There were 95 (79.2%) male patients and 25 (20.8%) female patients who were enrolled in the study. Forty-nine (51.2%) males had microalbuminuria while 46 (48.8%) did not. Eleven (44%) females had microalbuminuria while 14 (56%) did not. There was no significant association that was identified between either gender and microalbuminuria (p = 0.5).

There were 45 (37.5%) patients with a prolonged QTc and 75 (62.5%) patients with a normal QTc. 35 (58.3%) patients with microalbuminuria had a prolonged QTc while 25 (41.7%) did not. Ten (16.7%) patients without microalbuminuria had a prolonged QTc while 50 (83.3%) did not. There was a statistically significant association that was identified between a prolonged QTC interval and microalbuminuria (p<0.001).

There were 37 (30.8%) patients with hypertension in the study, while 83 (69.2%) patients were normotensive. Twenty-four (40%) patients with microalbuminuria had hypertension while 36 (60%) did not. Thirteen (21.7%) patients without microalbuminuria had hypertension while 47 (78.3%) did not. There was a statistically significant association that was identified between the presence of hypertension and microalbuminuria (p=0.03). The median duration of diabetes in the study was 10 years. The median duration of diabetes in the study group was 11.5 years, while the median duration of diabetes in the control group was 8 years. There was a statistically significant association that was identified between microalbuminuria and a longer duration of diabetes (p=0.034).

The mean HbA1c in the study was 8.4 ± 1.8%. The mean HbA1c in the study group was 9±1.9%, while the mean HbA1c in the control group was 7.8±1.6%. There was a statistically significant association that was identified between higher HbA1c values and microalbuminuria (p<0.001). The mean serum creatinine in the study was 1 ± 0.37 mg/dL. The mean serum creatinine in the study group was 1.1±0.4 mg/dL, while the mean serum creatinine in the control group was 0.9±0.3 mg/dL. There was a statistically significant association that was identified between higher serum creatinine and microalbuminuria (p=0.011). The results are summarised in Table 1.

**Table 1:** Summary of the results of the study. ^1^Expressed as mean ± standard deviation, ^2^Expressed as median HbA1C: haemoglobin A1C

Parameter	Microalbuminuria (study group) n= 60	Normoalbuminuria (control group) n=60	p-value
Age ^1^(years)	59.9±12.2	56.2±9.6	0.065
Male gender	49 (51.2%)	46 (48.8%)	0.5
Female gender	11 (44%)	14 (56%)	
Prolonged QTc	35 (58.3%)	10 (16.7%)	<0.001
Hypertension	24 (40%)	13 (21.7%)	0.03
Duration of diabetes ^2^(years)	11.5	8	0.034
HbA1C ^1^(percentage)	9±1.9	7.8±1.6	<0.001
Serum creatinine (mg/dL)	1.1±0.4	0.9±0.3	0.011

## Discussion

There is evidence that QTc interval prolongation and microalbuminuria are both possible complications of type 2 diabetes and that they may be related. QTc interval prolongation is a measure of the duration of ventricular repolarisation on ECG and is associated with an increased risk of arrhythmia, sudden cardiac death, and cardiovascular death. Microalbuminuria is an early marker of kidney disease in diabetic patients. We found a significant association between prolonged QTc intervals and the presence of microalbuminuria in our patients.

Several studies have examined the association between the prolongation of QTc interval and microalbuminuria in patients with type 2 diabetes. A study by Kobayashi et al. investigated the relationship between the QTc interval and microalbuminuria in 80 T2DM patients [12]. The study found that a patient with microalbuminuria also had a significantly longer QTc interval when compared to a patient without microalbuminuria. In addition, a study by Kumar et al. examined the association of QTc interval, microalbuminuria, and inflammatory markers in patients with T2DM and found that patients with microalbuminuria had significantly longer QTc intervals compared with those without microalbuminuria, as well as an association between prolonged QTc with a longer duration of T2DM [13]. The authors suggested that QTc interval prolongation could be a useful marker for identifying patients at high risk of developing microalbuminuria and diabetic nephropathy.

Our study also demonstrated a significant association between a longer duration of T2DM, higher HbA1c values, and the presence of microalbuminuria. This has been well-established in previous studies as well [14]. In summary, there is evidence of an association between QTc interval prolongation and the presence of microalbuminuria in T2DM. This makes the QTc interval prolongation a useful marker for identifying patients at increased risk of developing microalbuminuria and diabetic nephropathy. However, further studies are needed to better understand the underlying mechanisms of this relationship and its clinical implications.

Our study had several limitations. Even though the sample size was calculated based on existing prevalence, the study did have a relatively small sample size, and larger multi-centre studies may be required to establish and replicate these results. The coronavirus disease 2019 (COVID-19) pandemic made recruiting patients to the study difficult and could have resulted in selection bias of the participants due to patients shielding and not visiting hospitals, leading to the selection of relatively healthier and younger patients. Finally, this was an observational study, and a longitudinal study may be better at assessing the impact of microalbuminuria on the other studied parameters.

## Conclusions

Our study shows that there is a positive correlation between the presence of microalbuminuria and QTc interval prolongation in patients with T2DM. Furthermore, our study shows a positive association between microalbuminuria and higher HbA1c levels and longer duration of diabetes, suggesting that early detection and treatment of microalbuminuria may improve glycaemic control and diabetes-related outcomes and help prevent complications.

Overall, our study highlights the importance of monitoring microalbuminuria and the QTc interval in patients with T2DM. The results of this study have important implications for the management and treatment of T2DM, helping to improve patient outcomes and reduce the risk of cardiovascular events.

## References

[1] Saeedi P, Petersohn I, Salpea P (2019). Global and regional diabetes prevalence estimates for 2019 and projections for 2030 and 2045: results from the International Diabetes Federation Diabetes Atlas, 9th edition. Diabetes Res Clin Pract.

[2] (2018). The increasing burden of diabetes and variations among the states of India: the global burden of disease study 1990-2016. Lancet Glob Health.

[3] Alicic RZ, Rooney MT, Tuttle KR (2017). Diabetic kidney disease: challenges, progress, and possibilities. Clin J Am Soc Nephrol.

[4] Duan J, Wang C, Liu D (2019). Prevalence and risk factors of chronic kidney disease and diabetic kidney disease in Chinese rural residents: a cross-sectional survey. Sci Rep.

[5] Unnikrishnan RI, Rema M, Pradeepa R, Deepa M, Shanthirani CS, Deepa R, Mohan V (2007). Prevalence and risk factors of diabetic nephropathy in an urban South Indian population: the Chennai urban rural epidemiology study (CURES 45). Diabetes Care.

[6] Ninkovic VM, Ninkovic SM, Miloradovic V (2016). Prevalence and risk factors for prolonged QT interval and QT dispersion in patients with type 2 diabetes. Acta Diabetol.

[7] Li X, Ren H, Xu ZR, Liu YJ, Yang XP, Liu JQ (2012). Prevalence and risk factors of prolonged QTc interval among Chinese patients with type 2 diabetes. Exp Diabetes Res.

[8] Mittal S, Yadav A, Saxena GN (2018). Predictive value and association between microalbuminuria and prolonged QTc interval in hypertensive and type 2 diabetes mellitus. IOSR-JDMS.

[9] Kerner W, Brückel J (2014). Definition, classification and diagnosis of diabetes mellitus. Exp Clin Endocrinol Diabetes.

[10] (2021). 2. classification and diagnosis of diabetes: standards of medical care in diabetes-2021. Diabetes Care.

[11] Rautaharju PM, Surawicz B, Gettes LS (2009). AHA/ACCF/HRS recommendations for the standardization and interpretation of the electrocardiogram: part IV: the ST segment, T and U waves, and the QT interval: a scientific statement from the American Heart Association Electrocardiography and Arrhythmias Committee, Council on Clinical Cardiology; the American College of Cardiology Foundation; and the Heart Rhythm Society. Endorsed by the International Society for Computerized Electrocardiology. J Am Coll Cardiol.

[12] Kobayashi S, Nagao M, Asai A, Fukuda I, Oikawa S, Sugihara H (2018). Severity and multiplicity of microvascular complications are associated with QT interval prolongation in patients with type 2 diabetes. J Diabetes Investig.

[13] Kumar SS, Palaniandavan S, Padma V (2021). Association between prolonged QTc interval and microalbuminuria in patients of type II diabetes mellitus. J Res Med Dent Sci.

[14] Varghese A, Deepa R, Rema M, Mohan V (2001). Prevalence of microalbuminuria in type 2 diabetes mellitus at a diabetes centre in southern India. Postgrad Med J.

